# Supplementary material for: A Luminescent Guest@MOF Nanoconfined Composite System for Solid-State Lighting
Source: Molecules. 2021 Dec 14;26(24):7583. doi: 10.3390/molecules26247583 (PMC8706567; doi:10.3390/molecules26247583)
Supplement: Supplementary file 1 [file molecules-26-07583-s001.zip › molecules-1491244-supplementary.pdf]

Supporting Information

for

A Luminescent Guest@MOF Nanoconfined Composite System for Solid-State Lighting

Tao Xiong[1], Yang Zhang[1], Nader Amin[2] and Jin-Chong Tan[1,*]

[1]Multifunctional Materials & Composites (MMC) Laboratory, Department of Engineering Science, University of Oxford, Parks Road, Oxford OX1 3PJ, UK.

[2]Department of Chemistry, University of Oxford, Mansfield Road, Oxford OX1 3TA, UK.

*Correspondence: jin-chong.tan@eng.ox.ac.uk

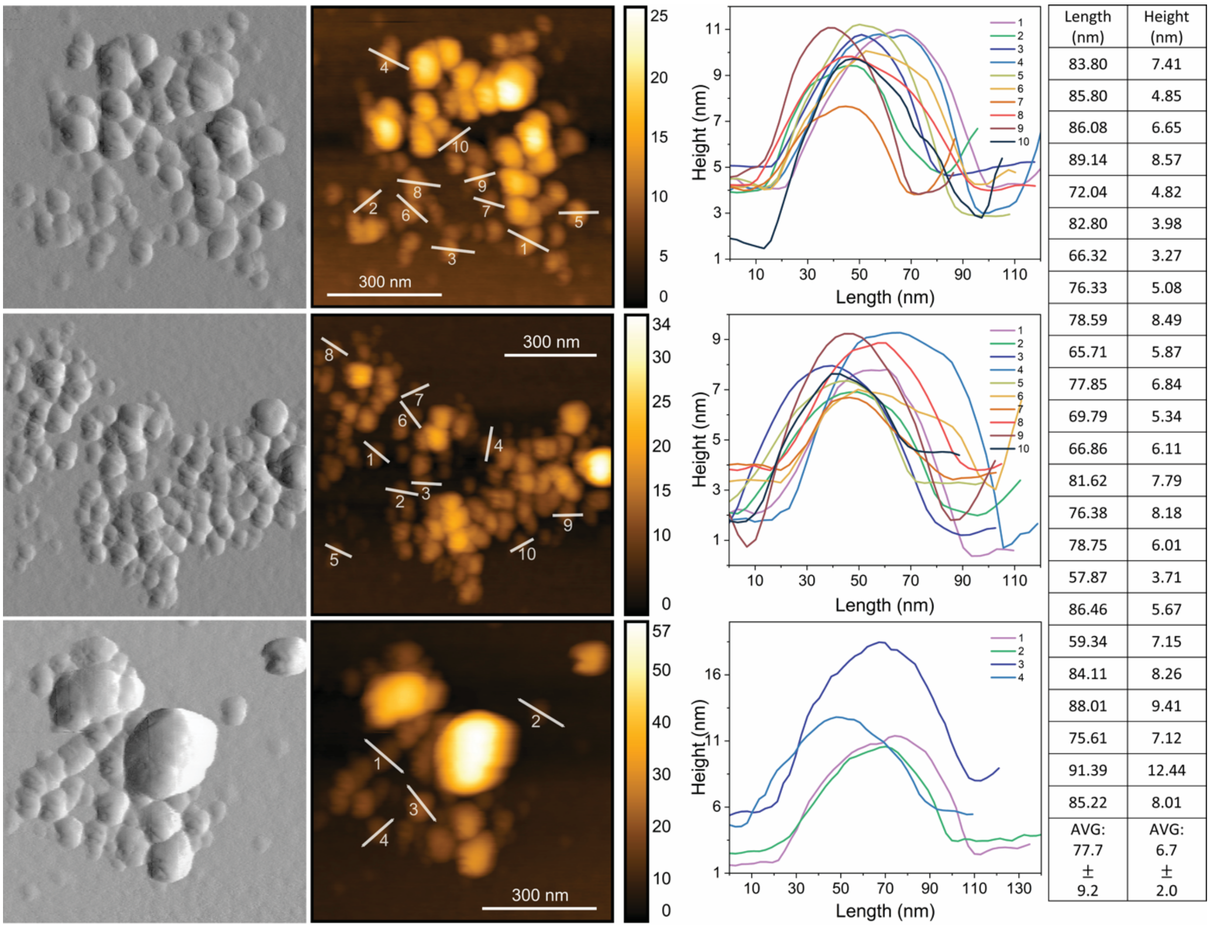

Length (nm)	Height (nm)
83.80	7.41
85.80	4.85
86.08	6.65
89.14	8.57
72.04	4.82
82.80	3.98
66.32	3.27
76.33	5.08
78.59	8.49
65.71	5.87
77.85	6.84
69.79	5.34
66.86	6.11
81.62	7.79
76.38	8.18
78.75	6.01
57.87	3.71
86.46	5.67
59.34	7.15
84.11	8.26
88.01	9.41
75.61	7.12
91.39	12.44
85.22	8.01
AVG: 77.7 ± 9.2	AVG: 6.7 ± 2.0

Figure S1. AFM topography images and height profiles of the 0.01 mM RhB@ZIF-8 nanocrystals. The width-to-height aspect ratio is about 10.

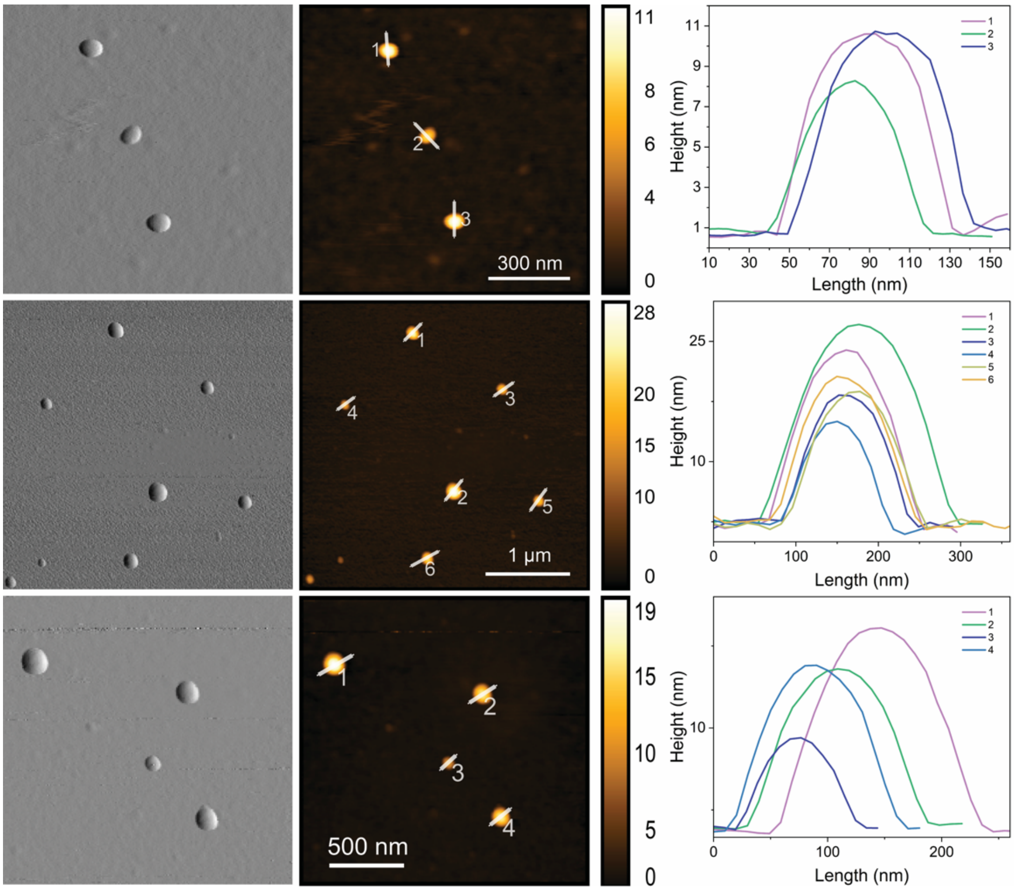

Length (nm)	Height (nm)
92.95	9.96
82.74	7.56
98.27	10.04
201.42	22.38
244.49	24.90
165.88	16.42
163.86	14.06
191.42	17.20
190.60	19.09
196.23	18.81
158.40	14.34
114.99	8.41
160.51	15.14
AVG: 158.6 ± 46.8	AVG: 15.3 ± 5.1

Figure S2. AFM topography images and height profiles of the 0.1 mM RhB@ZIF-8 nanocrystals.

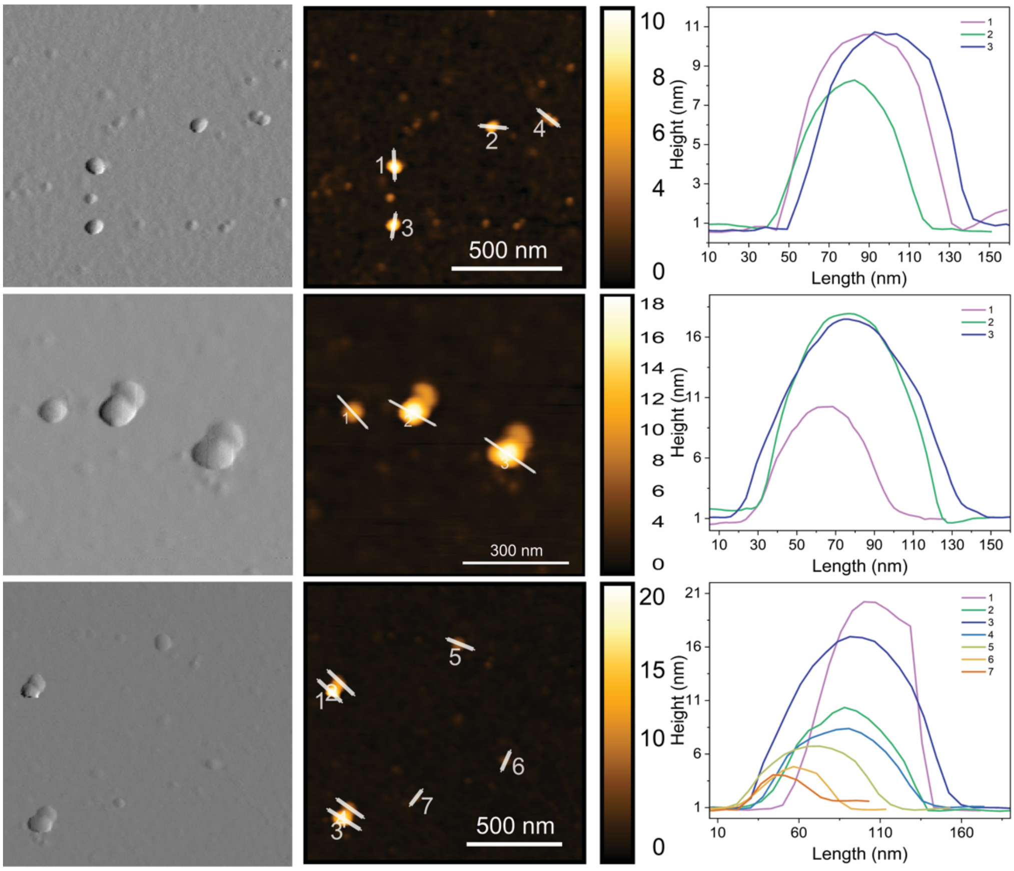

Length (nm)	Height (nm)
86.94	9.52
101.02	17.31
129.60	16.33
92.21	9.52
98.36	17.31
132.24	16.37
92.85	19.16
110.00	9.61
136.97	16.00
113.31	7.41
101.78	5.84
70.83	3.93
58.81	3.27
AVG: 101.9 ± 22.1	AVG: 11.7 ± 5.4

Figure S3. AFM topography images and height profiles of the 1 mM RhB@ZIF-8 nanocrystals.

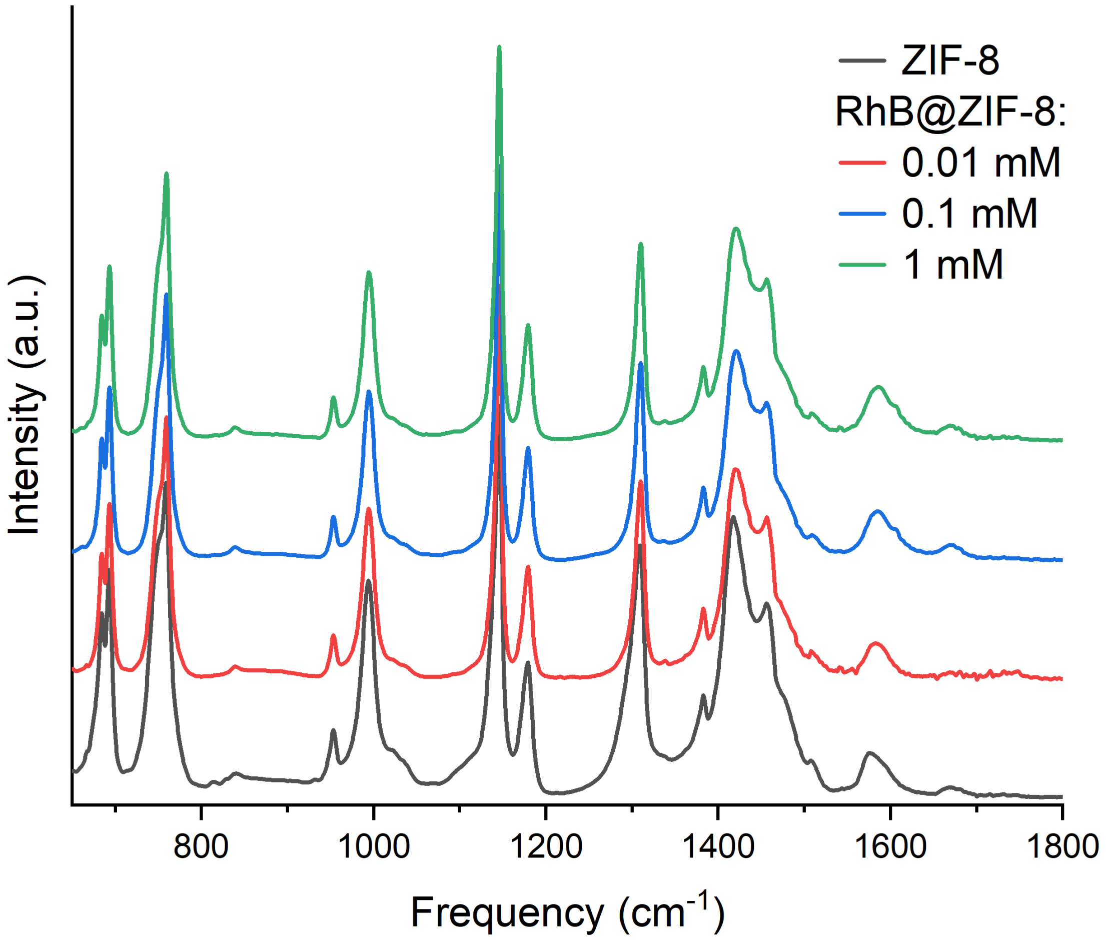

Figure S4. Comparison of the FTIR spectra of the (pristine) ZIF-8 and the RhB@ZIF-8 samples containing guest loadings of 0.01, 0.1, and 1 mM.

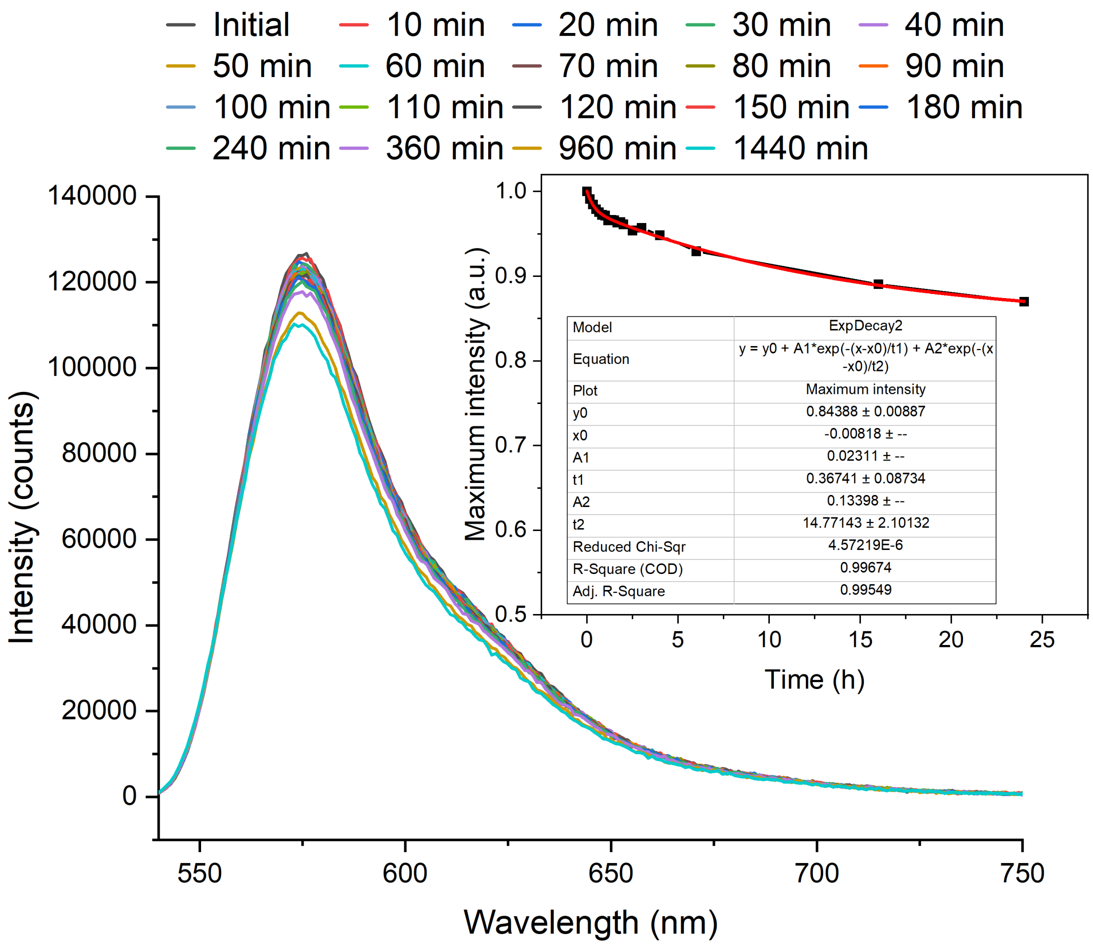

Figure S5. Sequential emission spectrum measurements of the 0.01 mM RhB@ZIF-8 samples under 450 nm excitation wavelength during a prolonged UV exposure time of up to 24 hours. Inset shows the UV-induced photodegradation as a function of exposure time under a continuous UV exposure generated by a Xenon lamp (FS-5 spectrofluorometer), with the maximum emission intensity of the initial measurement normalized to 1. The red line is a two-exponential decay curve fit of the experimental data, yielding an adjusted $R^2 = 0.99549$; all the other fitted parameters are shown in the table inset.

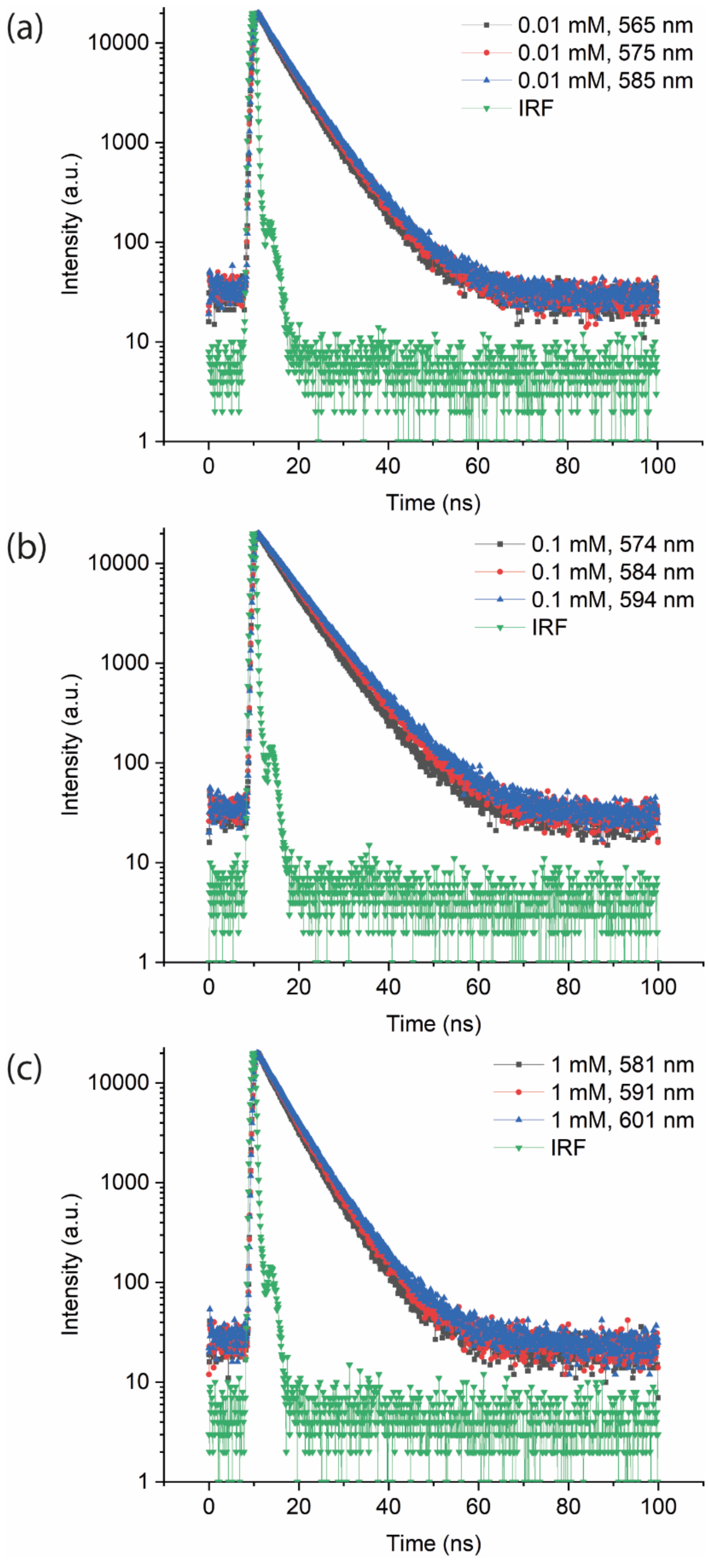

Figure S6. Luminescence lifetime decay curves for the RhB@ZIF-8 samples (0.01, 0.1, 1 mM) measured at three different excitation wavelengths each, together with their respective instrumental response function (IRF). The y-axes are in $\log_{10}$ scale. The fitted time constants ($\tau_{1\text{-}3}$) are presented in Table S1.

Table S1. Values of lifetime constants (τ_i) and fractional contributions (a_i) of the corresponding emission decay components of the RhB@ZIF-8 samples upon excitation at 362.5 nm, obtained from a multi-exponential fitting function, $I(t) = \sum_i a_i e^{-t/\tau_i}$, where $I(t)$ is the photon counts. The shortest lifetime component (τ_1 = 2 ns) is assigned to surface species, the intermediate lifetime component (τ_2 = 4-5 ns) assigned to aggregates, and the largest lifetime (τ_3 = 7-8 ns) assigned to RhB monomers. All χ^2 values are less than 1.3, thus signifying a good quality of fit to the experimental decay data shown in Figure S6.

**RhB@ZIF-8 (RhB concentration employed at synthesis, mM)**	***λ* nm**	**τ_1 ns**	**a_1 %**	**τ_2 ns**	**a_2 %**	**τ_3 ns**	**a_3 %**	**χ^2**
**0.01 mM**	565			3.9	40.83	7.0	59.17	1.266
	575			3.9	32.02	7.0	67.98	1.292
	585			3.9	25.14	7.0	74.86	1.237
**0.1 mM**	574			4.9	50.13	7.9	49.87	1.248
	584			4.9	32.18	7.9	67.82	1.201
	594			4.9	19.51	7.9	80.49	1.161
**1 mM**	581	2.0	6.05	4.6	64.31	7.1	29.65	1.224
	591	2.0	2.09	4.6	59.63	7.1	38.29	1.152
	601	2.0	1.12	4.6	51.26	7.1	47.62	1.104

Solution ^{1}H NMR spectroscopy of RhB@ZIF-8

Samples for NMR were dissolved in a solution composed of 500 µL methanol-d4 and 50 µL DCl / D_2O (35 wt%). All NMR spectroscopy was done at 298 K using a Bruker Avance NEO spectrometer operating at 600 MHz, equipped with a BBO cryoprobe. Data was collected using a relaxation delay of 30s, with 128k points and a sweep width of 19.8 ppm, giving a digital resolution of 0.18 Hz. Data was processed using Bruker Topspin with a line broadening of 1 Hz and 2 rounds of zero-filling.

Peaks were integrated using global spectral deconvolution in the MestReNova software package. For rhodamine B (RhB) the doublet at 8.33 ppm was used, which corresponds to a single aromatic proton on the benzoic acid group. For methyl imidazole (i.e. mIm linker of the ZIF-8 host), the singlet at 7.42 ppm was used, which corresponds to the 2 protons of the imidazole ring.

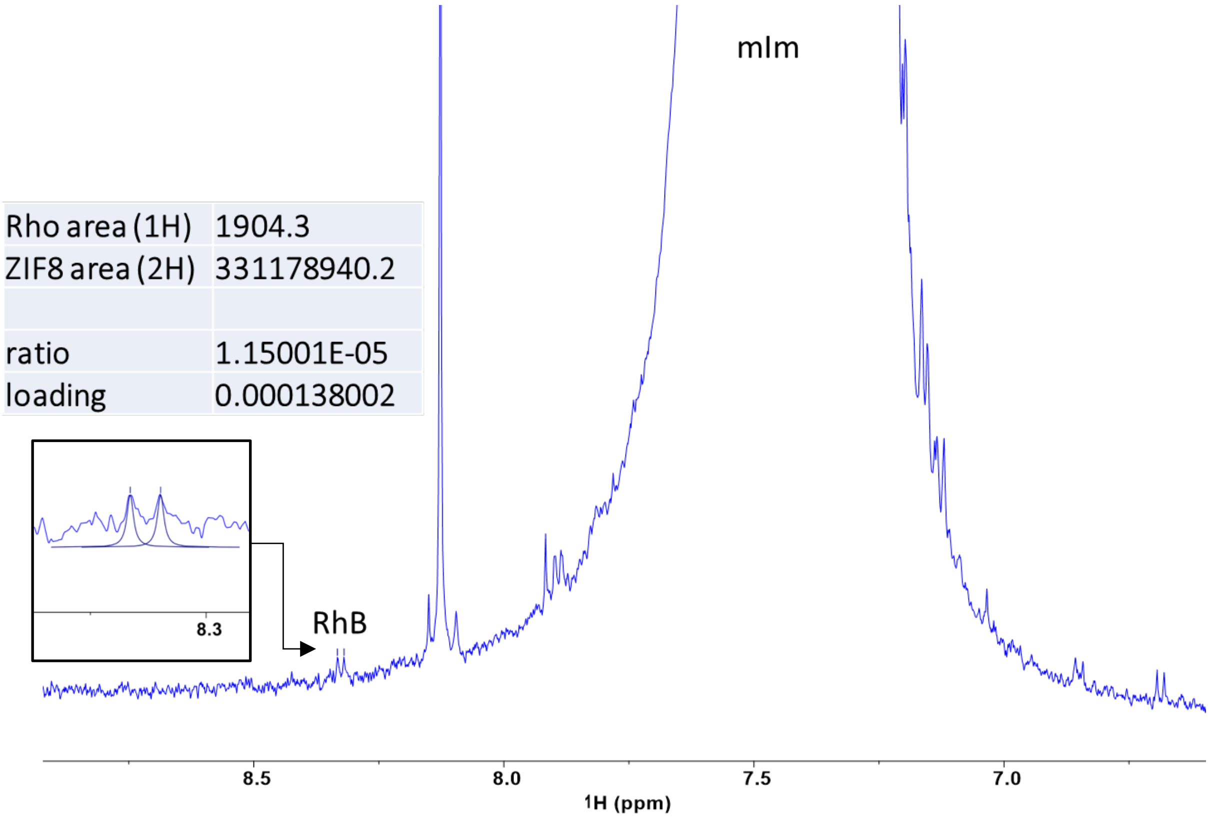

Figure S7. Solution ^{1}H NMR of 0.01 mM RhB@ZIF-8 where the guest/host peaks used for integration are indicated as RhB and mIm, respectively. The inset shows the integration of the the doublet at 8.33 ppm corresponding to a single aromatic proton on the benzoic acid group of RhB. The guest loading calculated is 1 RhB for every 7246.3 cages.

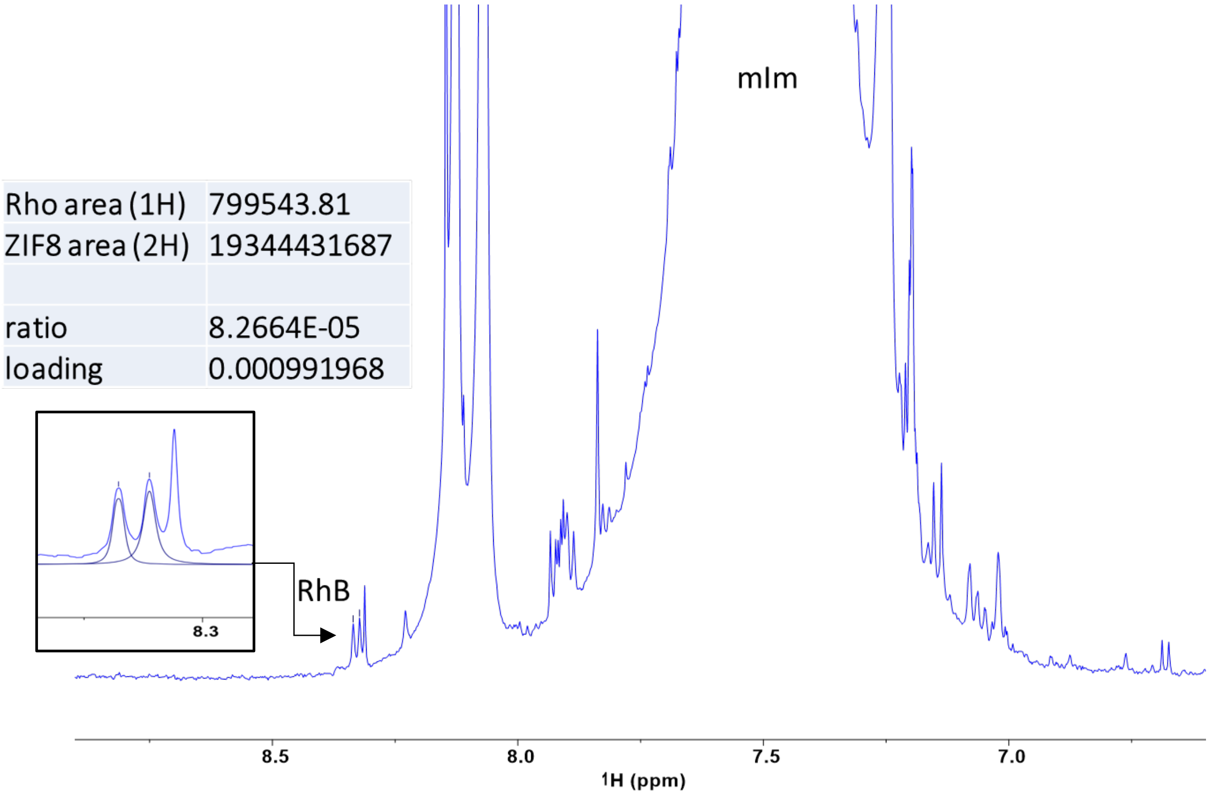

Figure S8. Solution ^{1}H NMR of 0.1 mM RhB@ZIF-8 where the guest/host peaks used for integration are indicated as RhB and mIm, respectively. The inset shows the integration of the the doublet at 8.33 ppm corresponding to a single aromatic proton on the benzoic acid group of RhB. The guest loading calculated is 1 RhB for every 1008.1 cages.

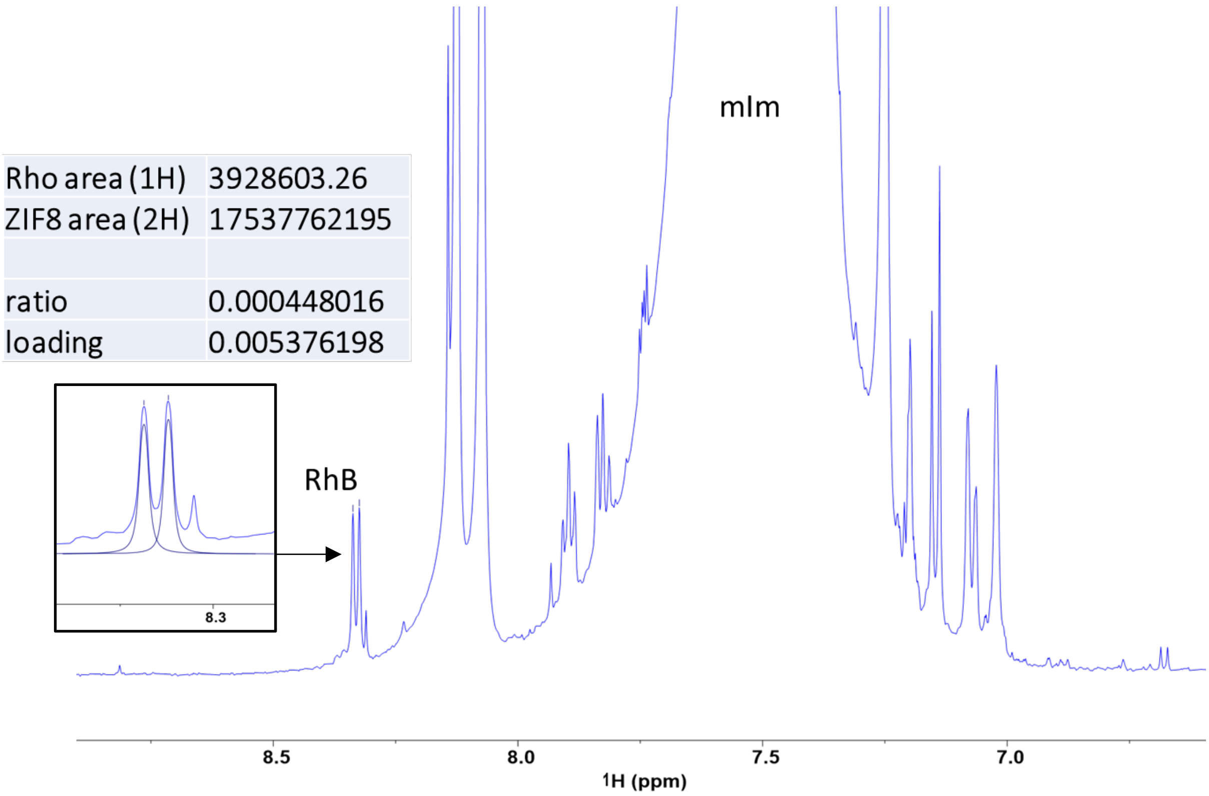

Figure S9. Solution ^{1}H NMR of 1 mM RhB@ZIF-8 where the guest/host peaks used for integration are indicated as RhB and mIm, respectively. The inset shows the integration of the the doublet at 8.33 ppm corresponding to a single aromatic proton on the benzoic acid group of RhB. The guest loading calculated is 1 RhB for every 186 cages.

Table S2. Detailed quantum yield (QY) data based on three measurements. $\overline{\mathrm{QY}}$ and σ_{QY} are the mean value and the standard deviation, respectively.

RhB@ZIF-8 (mM)	QY1 (%)	QY2 (%)	QY3 (%)	$\overline{QY}$ (%)	σ_{QY} (%)
0.01	99.58	98.20	99.24	99.00	0.72
0.1	94.47	94.13	94.03	94.21	0.23
1	71.06	72.38	70.92	71.45	0.81

Table S3. The quantum yield (QY) of RhB-related materials reported in the literature in comparison with the current work.

System	QY (%)	References
RhB/PVAc; RhB/PMMA	3.22 – 25.2	[1]
RhB@AuNP	1	[2]
RhB/sol-gel silica	37.4	[3]
RhB solutions	30 – 66	[4]
RhB@ZIF-71/PVDF electrospun fibers 1 wt%, 8 μL/min	92 ± 0.5	[5]
RhB@ZIF-8 (0.01 mM)	99 ± 0.7	This work

References

(1) Ahmed, R. M.; Saif, M., Optical Properties of Rhodamine B Dye Doped in Transparent Polymers for Sensor Application. *Chin. J. Phys.* **2013,** 51, 511-521.

(2) Stobiecka, M.; Hepel, M., Multimodal Coupling of Optical Transitions and Plasmonic Oscillations in Rhodamine B Modified Gold Nanoparticles. *Phys. Chem. Chem. Phys.* **2011,** 13, 1131-1139.

(3) Khader, M. A., Lasing Characteristics of Rhodamine B and Rhodamine 6G as a Sensitizer in Sol–Gel Silica. *Opt. Laser Technol.* **2008,** 40, 445-452.

(4) Sagoo, S. K.; Jockusch, R. A., The Fluorescence Properties of Cationic Rhodamine B in the Gas Phase. *J. Photochem. Photobiol. A* **2011,** 220, 173-178.

(5) Zhang, Y.; Tan, J. C., Electrospun Rhodamine@MOF/Polymer Luminescent Fibers with a Quantum Yield of Over 90%. *iScience* **2021,** 24, 103035.
